# Comparative Proteomic and Biochemical Analyses Reveal Different Molecular Events Occurring in the Process of Fiber Initiation between Wild-Type Allotetraploid Cotton and Its *Fuzzless-Lintless* Mutant

**DOI:** 10.1371/journal.pone.0117049

**Published:** 2015-02-20

**Authors:** Yuan Yao, Bing Zhang, Chun-Juan Dong, Ying Du, Lin Jiang, Jin-Yuan Liu

**Affiliations:** Laboratory of Plant Molecular Biology, Center for Plant Biology, School of Life Sciences, Tsinghua University, Beijing, China; National Key Laboratory of Crop Genetic Improvement, CHINA

## Abstract

To explore lint fiber initiation-related proteins in allotetraploid cotton (*Gossypium hirsutum* L.), a comparative proteomic analysis was performed between wild-type cotton (Xu-142) and its *fuzzless-lintless* mutant (Xu-142-*fl*) at five developmental time points for lint fiber initiation from -3 to +3 days post-anthesis (dpa). Using two-dimensional gel electrophoresis (2-DE) combined with mass spectrometry (MS) analyses, 91 differentially accumulated protein (DAP) species that are related to fiber initiation were successfully identified, of which 58 preferentially accumulated in the wild-type and 33 species in the *fl* mutant. These DAPs are involved in various cellular and metabolic processes, mainly including important energy/carbohydrate metabolism, redox homeostasis, amino acid and fatty acid biosynthesis, protein quality control, cytoskeleton dynamics, and anthocyanidin metabolism. Further physiological and biochemical experiments revealed dynamic changes in the carbohydrate flux and H_2_O_2_ levels in the cotton fiber initiation process. Compared with those in the *fl* mutant, the contents of glucose and fructose in wild-type ovules sharply increased after anthesis with a relatively higher rate of amino acid biosynthesis. The relative sugar starvation and lower rate of amino acid biosynthesis in the *fl* mutant ovules may impede the carbohydrate/energy supply and cell wall synthesis, which is consistent with the proteomic results. However, the H_2_O_2_ burst was only observed in the wild-type ovules on the day of anthesis. Cotton boll injection experiments in combination with electron microscope observation collectively indicated that H_2_O_2_ burst, which is negatively regulated by ascorbate peroxidases (APx), plays an important role in the fiber initiation process. Taken together, our study demonstrates a putative network of DAP species related to fiber initiation in cotton ovules and provides a foundation for future studies on the specific functions of these proteins in fiber development.

## Introduction

Cotton fiber is the most important natural textile fiber in the world. In cultivated cotton, such as *Gossypium hirsutum* L., the outer epidermal layer cells of the ovular differentiate into fiber primordial during the initiation stage from -3 to +3 days post-anthesis (dpa) and begin to elongate rapidly after fertilization. After a period of elongation lasting for 15 to 20 days, massive amounts of cellulose are deposited in the secondary cell wall, after which the fibers desiccate to form the mature lint fiber that could be used for spinning into yarn [1–4]. However, only approximately 30% of epidermal cells on the ovular surface differentiate into fiber primordia during the first round of fiber initiation [4]. Considering that limits on the number of cells that differentiate into lint fiber initials will restrict the yield, great endeavors were made to uncover the regulatory mechanisms underlying fiber initiation at the different molecular levels of transcriptome, proteome and individual genes [2, 3, 5–7].

In the past decade, proteomics has attracted more and more attention due to its advantages in providing abundant credible information regarding changes in protein abundance and post-translational modification, which are essential for understanding the physiological function of proteins [2, 8, 9]. In our previous study, an efficient modified protein extraction method for proteomic analysis was established for developing cotton fibers and was successfully applied in two-dimensional gel electrophoresis and mass spectrometry identification (2-DE/MS) [10]. Based on this improved technology, several proteomic studies were performed to understand the molecular events of cotton fiber development [11–14]. Yang et al. reported a dynamic proteome profile of cotton fibers during the elongation stage (5–25 dpa) and first depicted a global protein network accompanying fiber elongation [11]. Zhang et al. further supplemented and analyzed these data, revealing that glycolysis is the most regulated metabolism pathway during the fiber elongation process [12]. Du et al. systematically analyzed the dynamic proteome profile of *fuzz* fiber initiation (1 to 9 dpa) and provided the evidence of important roles of GA and H_2_O_2_ in the *fuzz* fiber initiation process [13]. Zhao et al. performed a comparative proteomic study between a short-lint fiber mutant (*Ligon lintless*, *Li*_*1*_) and its wild-type and identified 81 fiber elongation-related proteins [14]. These proteomic studies collectively indicated that most of the differentially accumulated proteins that are specific for fiber development were related to energy/carbohydrate metabolism, protein turnover, cytoskeleton dynamics, cellular responses and redox homeostasis [2, 11–14]. The information that was acquired from these proteomic analyses, together with the functional studies of individual proteins, shed new light on the understanding of fiber morphogenesis. However, most of the molecular mechanisms underlying cotton fiber development are still largely unclear, especially for early fiber cell protrusion.

In cultivated species of cotton plants, several spontaneous mutants for fiber initiation have been discovered, some of which have been subjected to genetic and molecular analyses [15–16]. Of these plants, a *fuzzless-lintless* (*fl*) seed mutant was reported to be a recessive mutation from *Gossypium hirsutum* cv. Xuzhou-142 (Xu-142). The Xu-142-*fl* plant shows no phenotypic difference from the wild-type except that its seeds lack both lint and fuzz fibers, making it a good genetic material for fiber initiation research [16]. Using a filter array containing 1536 cDNAs, Ji et al. compared the gene transcription profiles of 5 dpa ovules between the Xu-142-*fl* mutant and its parental wild-type and identified ten genes that were preferentially transcribed in cotton fibers [17]. ESTs representing whole ovules (from -3 to +3 dpa) have also been analyzed and are highly enriched with genes encoding putative transcription factors, such as MYB and WRKY, and genes encoding predicted phytohormonal regulators that are involved in the auxin, brassinosteroid (BR), gibberellic acid (GA), abscisic acid (ABA) and ethylene signaling pathways [18, 19]. Furthermore, cotton homologs that are related to *MIXTA*, *MYB5*, *GL2* and eight genes in the auxin, BR, GA and ethylene pathways were induced during fiber initiation in the wild-type but were repressed in the naked seed mutant [20]. Using a 2-DE-based comparative proteomics method, Liu et al. analyzed and compared the proteome profiles of -3 and 0 dpa cotton ovules between the wild-type (Xu-142) and *fuzzless-lintless* mutant (Xu-142-*fl*) and found that 46 proteins showed significant differences, including many ROS-related and stress-responsive proteins, suggesting that ROS generation increased in Xu-142-*fl* mutant coupled with a decreased stress response, lowered ROS scavenging capability, lowered carbohydrate metabolism, and down-regulated post-transcriptional and post-translational regulation [21]. In combination with the transcriptomics research, proteomics analyses greatly improved our understanding of the fiber initiation defect of the Xu-142-*fl* mutant cotton.

In this study, a precise 2-DE-based comparative proteomic analysis was performed to systematically profile the dynamic proteomes of allotetraploid cotton ovules between wild-type cotton (Xu-142) and its *fuzzless-lintless* mutant (Xu-142-*fl*) during the entire process of lint fiber initiation at five distinct time points of -3, -1, 0, +1 and +3 dpa. Of the 1,800 stained protein spots that were reproducibly detected in each gel image, a total of 91 protein spots were found to be differentially accumulated in wild-type/mutant allotetraploid cotton ovules with a greater than 1.5-fold abundance dynamics between any two time points of the initiation process. The MS identification of 91 differentially accumulated proteins (DAPs) combined with their abundance changes as well as the results that were obtained from biochemical analyses revealed a series of different molecular events that are potentially involved in cotton fiber initiation processes. Most importantly, in contrast to previous proteomics studies, our analyses collectively revealed that the ROS burst at 0 dpa was prominent in Xu-142 wild-type cotton ovules but not in those of the *fl* mutant, suggesting that a high level of ROS is important for proper fiber initiation, which was confirmed through an *in vivo* addition experiment of H_2_O_2_ in unfertilized cotton ovules. The results not only explained the temporal-spatial co-regulation in the current functional genomics datasets but also helped to depict a full picture of the regulatory networks of essential proteins for cotton fiber initiation.

## Results and Discussion

### Dynamic proteome changes in cotton ovules during the initiation process of fiber cells

**Fig 1:**
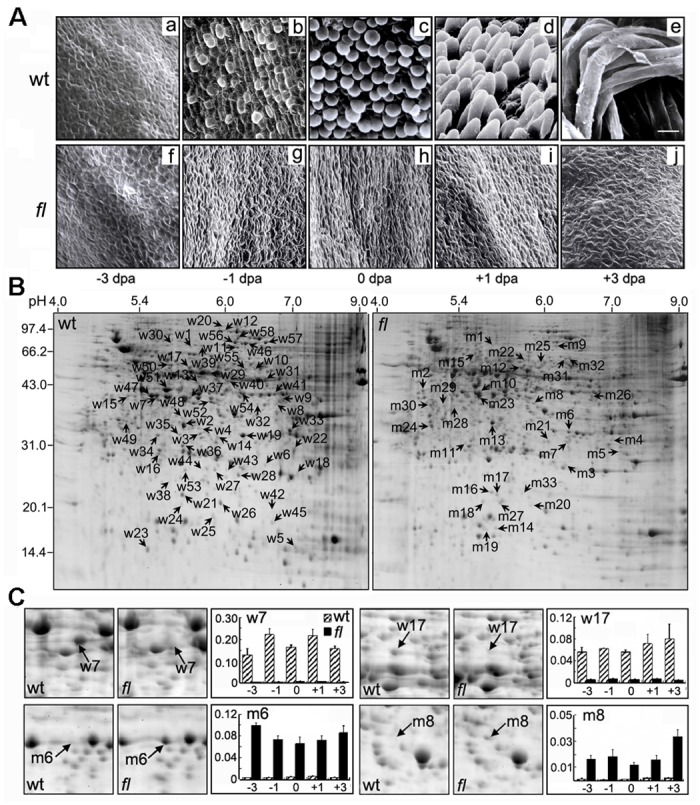
Scanning Electron Microscopy (SEM) images and representative 2-DE maps of the total proteins that were extracted from the cotton ovules of Xu-142 wild-type (wt) and its fiber-less mutant (*fl*). (A) SEM images of the cotton ovular surfaces in the fiber initiation stages at -3 (a, f), -1 (b, g), 0 (c, h), +1 (d, i) and +3 (e, j) dpa from wt (a-e) and *fl* mutant (f-j). The scale bar is 30 μm for all of the images. (B) The representative 2-DE maps of the total proteins from +3 dpa cotton ovules of the wt and *fl* mutant. A total of 91 differentially accumulated protein spots (DAPs) were displayed on the gels. The spots that predominately accumulated in the wild-type (w1–w58) are presented on the “wt” gel, while those that preferentially accumulated in the mutant (m1–m33) are shown on the “fl” gel. (C) Representative differentially accumulated protein spots and their volume% value in the 2-DE gels of the wt and *fl* mutant cotton ovules. The volume% value (y-axes) of each spot was calculated by three independent experiments.

Protrusions of fiber cells above the ovular surface during the initiation process from -3 to +3 dpa in cotton ovules were examined using scanning electron microscopy (SEM). As shown in Fig. 1-A-a to 1-A-e, most of the cells in the wild-type that were destined to develop into lint fiber cells had already started producing fiber initials on the day before anthesis (-1 dpa) (Fig. 1-A-b), and morphologically distinct fiber initials (Fig. 1-A-d) began to undergo rapid cell growth from +1 dpa and reached approximately 3 mm long at +3 dpa (Fig. 1-A-e). In contrast, the possible protodermal cells in the Xu-142-*fl* mutant remained unchanged, and no fiber initials were observed on the seed epidermis between -3 and +3 dpa as shown in Fig. 1-A-f to 1-A–j. The lack of fiber initials on the Xu-142-*fl* mutant ovules makes this mutant a unique genetic material for the identification of important proteins that might correlate with fiber cell initiation.

To explore lint fiber initiation-related proteins in allotetraploid cotton ovules, a 2-DE-based proteomic analysis was performed to profile the ovular proteomes of five important time points (-3, -1, 0, +1, and +3 dpa) during the fiber initiation process between wild-typeXu-142 and its mutant Xu-142-*fl*. Using the modified phenol extraction protocol [10] in combination with silver blue staining [22], approximately 1800 stained spots were reproducibly detected by image analysis on each 2-DE gel across pH 3–10 (Fig. 1-B). Through a spot-to-spot comparison and quantitative analysis, a total of 91 protein spots (the number of each spot is shown in Fig. 1-B) were found to have significant changes (*p-value*<0.05) between the wild-type and its mutant (S1 Table). The detailed abundance variance information of these 91 DAPs is listed in S2 Table. Of these spots, 58 were predominantly accumulated in the wild-type (numbered from w1–w58, Fig. 1-B, left), whereas 33 spots were displayed preferentially in the mutant (numbered from m1–m33, Fig. 1-B, right).

Among these 91 protein spots, most had a greater than 1.5-fold change in abundance during the fiber initiation process between the wild-type and its mutant, and 49 of these spots displayed a more than 5-fold change (S2 Table). Furthermore, a gel-based proteome profiles comparison indicated that 8 spots (including w7 and w17) could only be detected on gels of the wild-type Xu-142, whereas 5 spots (including m6 and m8) showed the opposite pattern, in which they were only present on the gel images for the mutant Xu-142-*fl* but could hardly be detected on the wild-type gels (Fig. 1-C, S2 Table). These results suggest that these spots might be specific to a certain phenotype and that some of them could be expected to serve as biomarkers for fiber initiation.

### Identification and molecular characterization of differentially accumulated proteins

**Fig 2:**
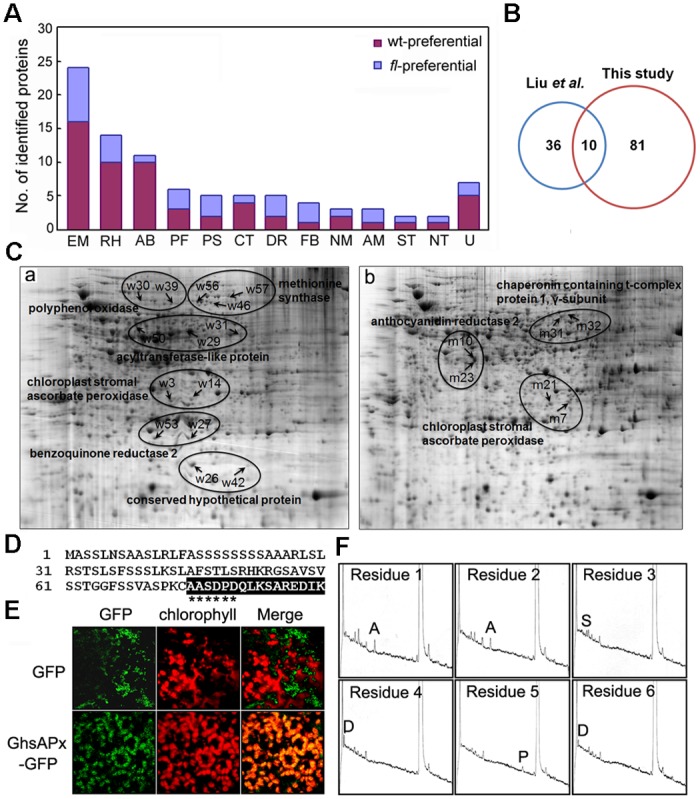
Functional categorization and isoform analyses of the 91 identified differentially accumulated proteins. (A) Functional classification of the 91 identified differentially accumulated proteins. Abbreviations: EM, energy/carbohydrate metabolism; RH, redox homeostasis; AB, amino acid biosynthesis; PF, protein folding and stabilization; PS, protein synthesis; CT, cytoskeleton; DR, defense responses; FB, fatty acid biosynthesis; NM, nucleotide metabolism; AM, anthocyanidin metabolism; ST, signaling transduction; NT, nucleocytoplasmic transport; and U, unclassified proteins. (B) Venn diagram analysis of the 91 identified DAPs in this study and the 46 DAPs that were reported in Liu et al. (C) Possible isoforms as revealed by the 2-DE proteomic analyses and MS identification. Protein species due to the same protein accession number were mapped on the 2-DE gels together to show the possible isoforms. (D) Signal peptide of the GhsAPx protein. The possible mature sequence after signal peptide cleavage is shown in the black box. (E) GhsAPx-GFP fusion protein was located in the chloroplasts of cotton seedling leaf cells. (F) N-terminal sequencing of the spot w3 GhsAPx protein. The first six amino acids of w3 were “AASDPD”, which is identical to those of m7.

In combination with mass spectroscopy (MALDI-TOF/TOF) analyses and the cotton EST database search, the 91 DAPs were all successfully identified and annotated, among which 73 DAPs were identified by PMF, and the remaining 18 DAPs were identified by MS/MS (S1 Table and S1 Fig.). Among the 91 identities, 84 (92%) were deposited in the current database as putative functional proteins, whereas 7 identities (spots w20, w21, w26, w42, w45, m1, and m18) were annotated as either hypothetical or unknown proteins. The 91 identities, which correspond to 69 unique proteins, could be classified into 12 major categories through online Gene Ontology analyses (http://www.geneontology.org) (Fig. 2-A and S1 Table). Most of the 58 identities that preferentially accumulated in the wild-type Xu-142 ovules were classified into the energy/carbohydrate metabolism (28%), redox homeostasis (17%), amino acid biosynthesis (17%), cytoskeleton (7%), and protein folding/stabilization (5%) categories, whereas the proteins that preferentially accumulated in the Xu-142-*fl* ovules were mainly included in the energy/carbohydrate metabolism (24%), redox homeostasis (12%), protein synthesis (9%), fatty acid biosynthesis (9%), and anthocyanidin metabolism (6%) categories (Fig. 2-A).

A Venn diagram analysis was then performed to compare the 91 DAPs that were identified in our study and 46 DAPs that were reported by another independent study [21]. Of the total 137 DAP spots, only 10 were detected in both studies, while the remaining 81 were first found to be related to fiber initiation (Fig. 2-B). The low overlap of these two datasets is understandable because 5 time points (-3, -1, 0, +1 and +3 dpa) surrounding the initiation process were analyzed in our study, whereas only two time points (-3 and 0 dpa) of the initiation process were chosen by another study [21], suggesting that the entire initiation process of cotton fibers is important for a comparative proteomics analysis to provide additional protein information regarding fiber initiation.

Our results also demonstrated that 30 identified proteins represent 11 unique proteins (*unipros*) (S1 Table). These identities could be divided into 2 categories. The first category included 3 protein species of acyltransferase-like protein (w29, w30 and w50), 2 protein species of benzoquinone reductase (w27 and w53), 3 protein species of 5-methyltetrahydropteroyltriglutamate/homocysteine methyltransferase (w46, w56 and w57), 2 protein species of anthocyanidin reductase 2 (m10 and m23), and 5 protein species of conserved hypothetical proteins (w20, w21, w26, w42 and w45). The identities of these unipros were only predominantly accumulated in either the wild-type or the *fl* ovules (Fig. 2-C-a/b and S1 Table). In contrast, the other 15 identities belonging to the second category were distributed on the gels of both the wild-type and *fl* mutant. These identities were comprised by 2 protein species of lactoylglutathione lyase (w2 and m13), 4 protein species of chloroplast stromal ascorbate peroxidase (w3, w14, m7 and m21), 2 protein species of dehydroascorbate reductase (w18 and m3), 2 protein species of the chloroplast precursor of ketol-acid reductoisomerase (w17 and m12), 3 protein species of the γ-subunit of chaperonin containing t-complex protein 1 (w43, m31 and m32) and 2 protein species of annexin 1 (w19 and m6) (Fig. 2-C and S1 Table). These distinct spots on the gels were annotated as “the different protein species of same protein”, which might be formed due to splice variants, signal peptide cleavage, post-translational modification or protein degradation. For example, chloroplast stromal ascorbate peroxidase (GhsAPx) contained 344 aa of full-length ORFs with a predicted molecular weight of approximately 37 kD. However, the observed Mw of the 4 DAP spots was 30–32 kD. A plastid transit signal peptide of approximately 70 aa was predicted in the N-terminus of this protein using TargetP software [23] (Fig. 2-D). A fusion GFP (green fluorescence protein) assay indicated that this signal peptide could drive the fused GFP protein to locate in the chloroplast of cotton leaves, as reflected by the merger of the green fluorescence of GFP with the red fluorescence of chlorophyll (Fig. 2-E). To ascertain the exact cleavage site of the signal peptide, Edman degradation was performed for the protein spots w3 and m7. As shown in Fig. 2-F, the first six amino acids of the two protein spots were determined to be “AASDPD”, which was completely identical to amino acids 75–80 of the full-length GhsAPx protein (Fig. 2-D). These results, combined with the observation of a smaller Mw, suggest that all 4 of the identified GhsAPx spots might be the mature protein form with the signal peptide cleaved, whereas the isoelectric point (pI) and the molecular weight (Mw) drifts that were displayed on the 2-DE gels by these 4 identified GhsAPx spots should be ascribed to post-translational modifications, such as reversible phosphorylation (Fig. 2-C). In fact, some different isoforms of the 40 identified DAPs that were revealed in the 2-DE-based proteomics analysis have been annotated by phosphorylation events in the cotton fiber elongation process [24].

Another example of multiple protein species of same protein is dehydroascorbate reductase (GhDHAR), which appeared as spots w18 and m3 on the gels. DHAR has been reported to undergo post-translational modification, such as S-glutathionylation in *Arabidopsis* [25]. The observed Mw of m3 was larger than that of w18 (S1 Table), and the difference (approximately 0.3 kD) was equivalent to the Mw of an S-glutathionylation modification (Gly-Cys-Glu, 305 Da), suggesting that GhDHAR underwent the S-glutathionylation modification in cotton ovules. However, further study was necessary to fully elucidate the existence of this modification and other possible modifications.

### Differentially accumulated proteins that are associated predominantly with active carbohydrate/energy metabolism

Fiber cell protrusions and their expansion above the ovular surface are achieved coordinately by cell wall loosening and cell wall biosynthesis [3]. The largest functional group (26 identities, 28.6% of the total) of the 91 DAPs was the protein species that were associated with carbohydrate/energy metabolism, which may provide energy and building blocks for the fiber initiation processes. These proteins could be divided into two distinct subgroups. The first subgroup was composed of some enzymes that participate in glycolysis, the citric acid cycle and the pentose phosphate pathway, including isocitrate dehydrogenase (w8), transketolase (w11), aconitase (w12), succinyl-CoA ligase beta subunit (w15) and triose phosphate isomerase (w16) (S1 Table). Most of these enzymes were predominantly accumulated in the ovules of the wild-type, suggesting that energic sugar catabolism is required for normal early fiber development. It is noteworthy that fructose-1,6-bisphosphatase (m8), a rate-limiting enzyme in gluconeogenesis, was down-regulated in the ovules of the wild-type from -3 to +3 dpa, suggesting that gluconeogenesis metabolism, especially the step of fructose-1,6-bisphosphate conversion to fructose 6-phosphate, is gradually inhibited as fiber initiation proceeds.

**Fig 3:**
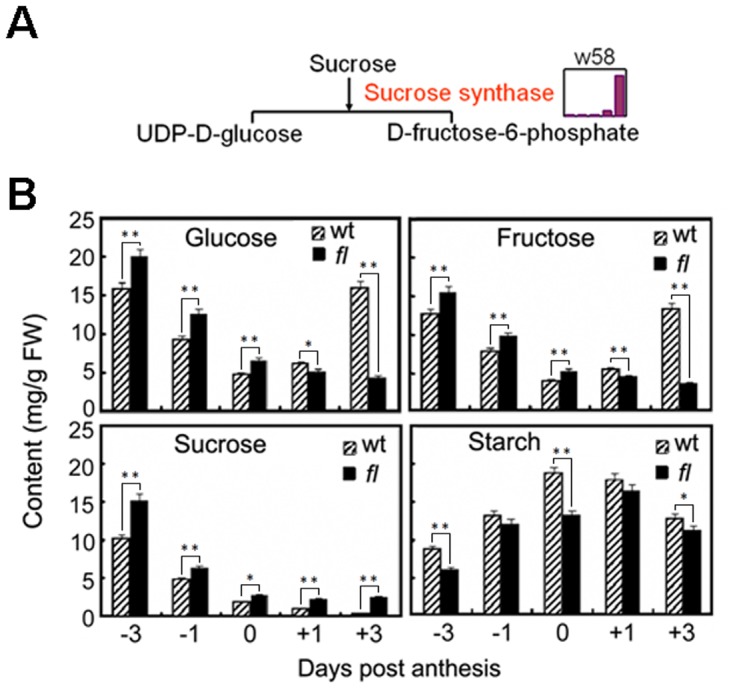
Carbohydrate metabolism is highly related to fiber initiation. (A) Sucrose synthase (w58) differentially accumulated in the wt and *fl* mutant cotton ovules. The bars indicate the log-transformed values of the fold-change ratios of differentially accumulated proteins at five stages. (B) Carbohydrate contents as determined by an enzymatic method. The results are the mean values from three independent experiments. Statistical analyses were performed using student’s T test. Abbreviations: *, p<0.05; **, p<0.01.

The second subgroup includes the proteins that seemingly serve for rapid cell expansion, such as sucrose synthase (w58), putative UDP-glucose dehydrogenase (w55) and reversibly glycosylated polypeptide (w52). In cotton plants, the sucrose that is produced by photosynthesis is transported to the cotton ovules, where the sucrose synthase preferentially converts sucrose into fructose and UDP-glucose [26, 27], which supplies the carbon skeleton and energy for cell expansion and cell wall cellulose synthesis. Ruan et al. reported that the suppression of sucrose synthase predominately expressed in cotton fibers led to fewer initials and a fibreless phenotype, most likely as a result of the reduction of its product fructose and UDP-glucose [8]. Our results support the conclusion that the protein abundance of sucrose synthase (w58) was lower in the *fl* mutant than that in the wild-type ovules (Fig. 3-A). UDP-glucose dehydrogenase and reversibly glycosylated polypeptides play major roles in the biosynthesis of cell wall polysaccharides [28, 29]. Protein abundance analyses indicated that these two proteins were not accumulated in wild-type Xu-142 ovules until +3 dpa, which is consistent with the requirement of rapid fiber elongation after +3 dpa.

The dynamic abundance variance of the enzymes participating in carbohydrate/energy metabolism may result in significant difference in the metabolism of common sugars between the Xu-142 wild-type and the *fl* mutant ovules. To verify this possibility, glucose, fructose and sucrose in early developing cotton ovules were qualified using an enzymatic method. As shown in Fig. 3-B, before blossom, all three types of sugar displayed similar declining patterns, and their contents in the *fl* mutant cotton ovules were slight higher than were those of wild-type ovules. After anthesis, sucrose still retained a similar decreasing pattern until +3 dpa, whereas the concentration variance of glucose and fructose were divergent in the wild-type and *fl* mutant cotton ovules. In the wild-type ovules, the concentration of the two monosaccharides gradually increased from 0 to +3 dpa and regained the peak of the -3 dpa ovules at +3 dpa. In contrast, the glucose and fructose contents in the *fl* mutant ovules were always low from 0 to +3 dpa. Similar results were obtained in GC-MS metabolite profiling experiments in that sucrose degradation and substrate level phosphorylation were down-regulated in the *fl* mutant cotton ovules [21]. Additionally, although the wild-type and *fl* mutant ovules possess a similar variance pattern as that of starch, which is often synthesized from sucrose and accumulates in developing seeds as an energy source for germination, the starch concentration in the wild-type was higher than that in the *fl* mutant at each time point. These results strongly suggest that *fl* mutant ovule utilized the sucrose with a low efficacy after anthesis, resulting in a state of monosaccharide and starch starvation compared to the wild-type ovule, which may not fulfill the demands of rapid fiber development.

### Differentially accumulated proteins that are related closely to protein turnover

**Fig 4:**
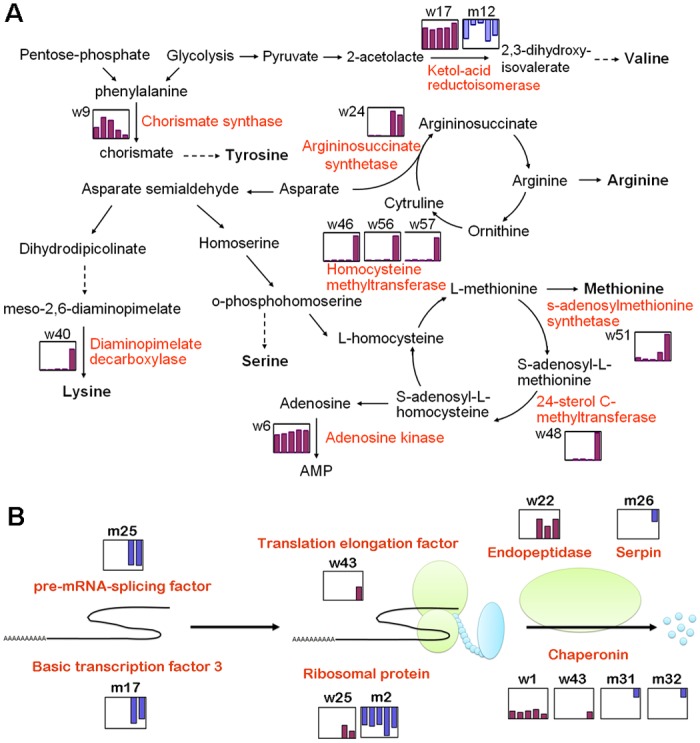
Overview of the differently accumulated proteins that are associated with amino acid biosynthesis and protein turnover. (A) Amino acid biosynthesis pathways indicating the enzymes that were identified as differentially accumulated in the wt and *fl* mutant cotton ovules. (B) Protein synthesis, folding and degradation pathways indicating the proteins that were identified as differentially accumulated in the wt and *fl* mutant cotton ovules. The bars indicate the log-transformed values of the fold-change ratios of differentially accumulated proteins at five stages, with those that accumulated in the wt charted in purple and those that accumulated in the *fl* mutant charted in blue.

As shown in S1 Table, a total of 11 DAPs (12% of the 91 identities) were related to amino acid biosynthesis, of which 10 were accumulated predominantly in wild-type cotton ovules during the initiation process, whereas only one was preferentially accumulated in the *fl* mutant ovules, suggesting that amino acid biosynthesis might be indispensable for fiber initiation. Among these DAPs, chorismate synthase (w9) catalyzes phenylalanine formation with the byproduct tyrosine, argininosuccinate synthase (w24) catalyzes the synthesis of arginine from aspartate, and diaminopimelate decarboxylase (w40) catalyzes the synthesis of lysine from aspartate [30–32]. It is noteworthy that three enzymes, s-adenosylmethionine synthetase (w51), 5-methyltetrahydropteroyltriglutamate/homocysteine methyltransferase as 3 protein species (w46, w56 and w57), and 24-sterol C-methyltransferase (w48), are involved in the methionine cycle and were obviously visible on the 2-DE gels of +3 dpa wild-type ovules, whereas these enzymes were hardly detected on the corresponding *fl* mutant ovules gels (S1 Table, Fig. 4-A). Adenosine kinase (w6), which catalyzes adenosine, the byproduct of methionine cycle, to adenosine monophosphate (AMP), was also only present on the gels of the wild-type ovules but not on those of the *fl* mutant. These observations indicate the importance of the methionine cycle in the normal fiber initiation of cotton ovules. As one of the major components of the primary wall of cotton fiber cells, pectin consists of partially methylated polygalacturonic acid units and is responsible for cell wall extensibility [33]. In *Arabidopsis*, the adenosine kinase deficiency was reported to lead to a lack of elongation of the primary shoot, the abnormal elongation of the stamen filaments and decreases hypocotyl and root elongation, which might be ascribed to less esterification of pectin due to the reduced methyltransferase [34]. Therefore, a high activity of the methionine cycle could promote the esterification of pectin by transmethylation reactions and could increase the expansibility of the cell wall, which is essential for the rapid elongation of fiber cells after initiation [35].

Furthermore, 11 DAPs were found to be associated with protein translation, folding and stabilization (Fig. 4-B). For the 5 identities functioning in protein biosynthesis, 2 identities were identified from wild-type ovules (S1 Table), including ribosomal protein S12 (w25) and eukaryotic translation elongation factor (w34), which are directly involved in the initiation and elongation of the newly synthesized peptide chains. The other 3 identities were preferentially present on the *fl* mutant ovules gels, including 40S ribosomal protein SA (m2), basic transcription factor 3 (m17) and pre-mRNA-splicing factor (m25), which are also essential elements of translation apparatus. These findings indicate that the protein synthesis rate might be different in the wild-type and *fl* mutant ovules. The other 6 identities were related to protein folding and stabilization, including heat shock protein 70 (w1), 20S proteasome α-subunit/endopeptidase/threonine-type endopeptidase (w22), serpin/putative serine protease inhibitor (m26) and the γ-subunit of chaperonin containing t-complex protein 1 with 3 protein species (w43, m31 and m32). Among these DAPs, chaperonin containing t-complex protein 1 (CCT1) acts as a molecular chaperone for tubulin, actin and most likely some other proteins [36]. In addition, the disassembly and reassembly of the cytoskeleton are responsible for directing cell expansion and are indispensable for cotton fiber development [37]. As shown in Figs. 1-B and 2-B, the possible degraded form of CCT1 (w43), which has a lower Mw, could only be detected on the gel of the wild-type ovules at +3 dpa, the jumping-off time point of cotton fiber rapid elongation, strongly suggesting that CCT1 might be an important regulator of fiber initiation.

### Differentially accumulated proteins that are preferentially involved in redox homeostasis

**Fig 5:**
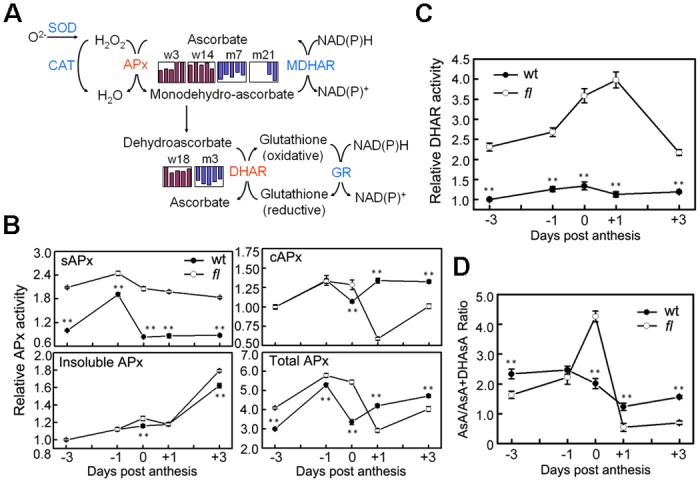
Ascorbate-glutathione cycle is differentially regulated during the fiber initiation process in the wt and *fl* mutant cotton ovules. (A) Ascorbate-glutathione cycle indicating the enzymes that were identified as differentially accumulated in wt and *fl* mutant cotton ovules. The bars indicate the log-transformed values of the fold-change ratios of differentially accumulated proteins at five stages, with those that accumulated in the wt charted in purple and those that accumulated in the *fl* mutant charted in blue. Abbreviations: SOD, superoxide dismutases; CAT, catalase; APx, ascorbate peroxidase; MDHAR, monodehydroascorbate reductase; DHAR, dehydroascorbate reductase; GR, glutathione reductase. (B) Relative activities of APx isozymes with different subcellular locations in early-developmental cotton ovules. The enzyme activity of wt ovules at -3 dpa was normalized to 1. (C) Relative activity of DHAR in early-developmental cotton ovules. The enzyme activities of wt ovules at -3 dpa were normalized to 1. (D) The ratio of ascorbate/dehydroascorbate in the ovules of the wt and *fl* mutant. The results are the mean values from three independent experiments. The results of *fl* mutant were compared with the wt using student’s T test. Abbreviations: *, p<0.05; **, p<0.01; sAPx, chloroplast stromal APx; and cAPx, cytosolic APx.

During the fiber initiation process, small toxic molecules, such as reactive oxygen species (ROS), are inevitably generated in many metabolic reactions, especially the carbohydrate and amino acid metabolism pathways [38]. The elimination of these toxic by-products in time is imperative to maintaining the intracellular homeostasis and is thus indispensable for effective fiber initiation. In most plant cells, the ascorbate-glutathione cycle is one of the major scavengers of ROS [39]. In this cycle, ascorbate is used as reducing reagent to detoxify H_2_O_2_ and other ROS. Oxidized ascorbate is further rescued by synergistic reactions that are catalyzed by ascorbate peroxidases (APx), monodehydroascorbate reductase (MDHAR), dehydroascorbate reductase (DHAR), and glutathione reductase (GR) [40]. Among the 91 DAPs, 4 and 2 spots that were distributed at different locations on the 2-DE gels of the wild-type and *fl* mutant ovules were identified as chloroplast stromal APx (sAPx, w3, w14, m7 and m21) and DHAR (w18 and m3), respectively (S1 Table, Fig. 5-A). As mentioned above, the different protein species might be formed through variance splicing and post-translational modification (S-glutathionylation), which could affect their molecular properties, such as stability, subcellular location and enzyme activity. Therefore, enzymatic assays of sAPx and DHAR were performed in the Xu-142 wild-type and *fl* mutant ovules to further characterize the functions of these DAPs.

Based on the different inactivation speeds in ascorbate-depleted solution, the activities of various subcellular APxs were first determined. As shown in Fig. 5-B, the activity of sAPx in the *fl* ovules was two-times higher than that of wild-type at each time point from -3 to +3 dpa, whereas the activity of cytosolic APx (cAPx) showed no significant difference before 0 dpa as this activity decreased in the *fl* mutant but not in the wild-type ovules (Fig. 5-B). Meanwhile, the activities of insoluble APx [APx localized at the thylakoid membrane (tAPx) and microbodies (mAPx)] were similar in the wild-type and *fl* mutant ovules (Fig. 5-B). Therefore, the difference in the total APx activity before anthesis could be almost ascribed to sAPx. Considering that cell fate determination for fiber initials occurs from -3 to 0 dpa, we suspect that the low activity of APx in the plastids of the ovules was a major determinant for cotton fiber initiation. The DHAR activity assay results further support this suspicion. As shown in Fig. 5-C, there was a sharp increase of DHAR activity in the *fl* mutant ovules at 0 and +1 dpa, the same as the abundance change of spot m3 in the *fl* mutant (S2 Table); however, no significant differences were found in the wild-type ovules. Furthermore, DHAR activities were much lower in the wild-type ovules than in the *fl* mutant ovules at each stage, resulting in less substrate ascorbate being recycled for use by APxs (Fig. 5-B). In agreement with these results, as shown in Fig. 5-D, the highest ratio of ascorbate/dehydroascorbate was observed in the *fl* mutant ovules at 0 dpa. This ratio did not significantly decrease in the wild-type ovules but significantly decreased in the *fl* mutant ovules after anthesis. As a good indicator of the degree of oxidative stress that is endured by plants, the ascorbate/dehydroascorbate ratio strongly suggests that the redox status is different in the wild-type and *fl* mutant ovules at early developmental stages.

**Fig 6:**
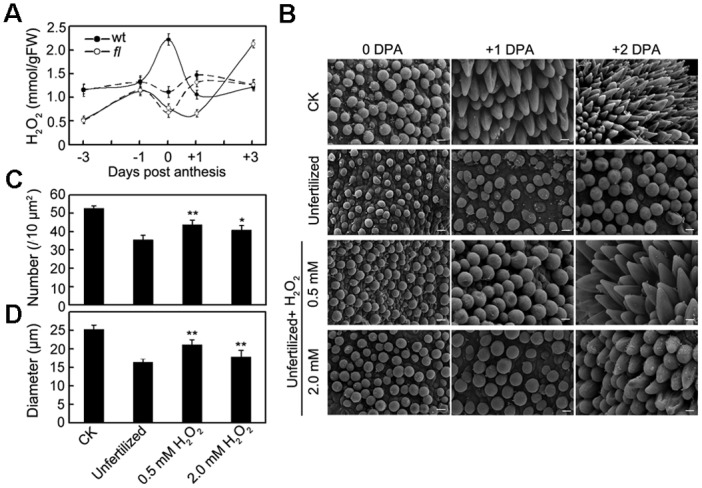
H_2_O_2_ could promote cotton fiber initiation. (A) H_2_O_2_ contents as determined in fertilized (solid lines) and unfertilized (dashed lines) cotton ovules of the wild-type and *fl* mutant. (B) Scanning electron micrographs of the ovules without or with fertilization blocked or the unfertilized ovules that were treated with 0.5 mM or 2.0 mM H_2_O_2_. The scale bar was 10 μm for all of the images. The number (C) and diameter (D) of the initiation bubbles on the surface of the ovules at 0 dpa were calculated. The results were compared with the unfertilized ovules using student’s T test. Abbreviations: *, p<0.05; **, p<0.01.

To determine the redox status of the early-developing wild-type and *fl* mutant ovules, the level of H_2_O_2_, which is the most prominent ROS molecule of plant cells, in the ovules at 5 time points was examined. As shown in Fig. 6-A, the H_2_O_2_ level of the wild-type ovules was much higher than that of the *fl* mutant ovules at -3 and-1 dpa. On the day of anthesis (0 dpa), the H_2_O_2_ level sharply increased and then rapidly decreased in the wild-type ovules, forming an inconceivable H_2_O_2_ burst. However, in the *fl* mutant ovules, the level of H_2_O_2_ decreased from 0 to +1 dpa and then rebounded and peaked at +3 dpa. The fertilization process seemed to be necessary for the formation of the H_2_O_2_ burst in the wild-type ovules because the burst of H_2_O_2_ disappeared when the androecium, stigma and style of flowers were removed on the evening of -1 dpa to block fertilization. Under unfertilized conditions, the wild-type and *fl* mutant ovules displayed a similar variance pattern as that of the H_2_O_2_ levels (Fig. 6-A, dashed lines).

The finding that the H_2_O_2_ burst was prominent in the wild-type ovules seems contradictory to results that were reported by Liu et al, who stated that the ROS level was higher in the *fl* mutant due to lowered ROS scavenging enzyme activities [21]. However, the ROS that are produced by an NADPH oxidase (RHD2) accumulated in *Arabidopsis* root hairs as it emerged, initially as a bulge and then elongated by tip growth. In contrast, the *rhd2* mutant was impaired in both ROS accumulation and root-hair elongation [41]. Similar results were also found in the developing pollen tubes. Mcinnis et al. indicated that high amounts of ROS, predominantly H_2_O_2_, accumulated in stigmatic papillae and could promote pollen tube extension [42]. Thus, the observation of an H_2_O_2_ burst in the wild-type but not in the *fl* mutant ovules indicates that the fertilization-dependent H_2_O_2_ burst could also promote fiber initiation. To test this suspicion, a series of *in vivo* ovule treatments were performed, and the ovular surfaces were analyzed by scanning electron microscopy (SEM). As shown in Fig. 6-B, distinct phenotypes could be observed for fertilized and unfertilized wild-type ovules. There were approximately 52.6 initiation bulbs per 0.01 mm^2^ on the fertilized ovular surface at 0 dpa, after which the fibers began elongation. When fertilization was blocked, fewer fibers could initiate (only approximately 67.8% compared to the fertilized ovules) (Fig. 6-C), and the bulb diameters were significantly smaller (Fig. 6-D). However, the reduced fiber initiation in unfertilized wild-type ovules could be partially rescued by the addition of 0.5 or 2.0 mM H_2_O_2_ (Fig. 6-B to D). These results indicate that the fertilization-dependent H_2_O_2_ burst on the day of anthesis is most likely one of the most important signals for cotton fiber initiation.

### A possible model for fiber cell initiation as revealed by comparative proteomics analyses

**Fig 7:**
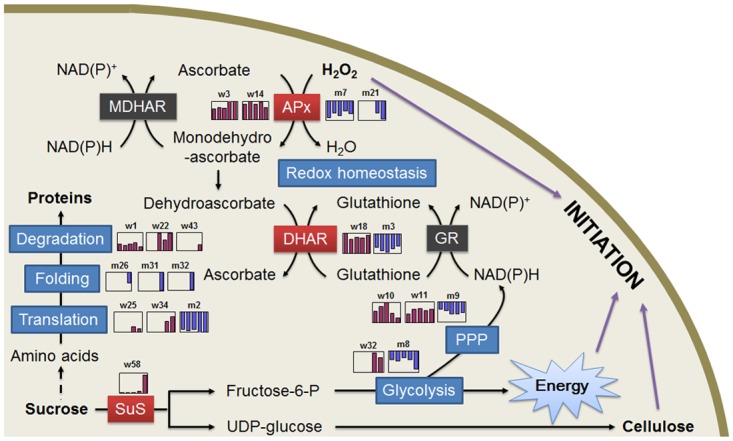
A putative network of differentially accumulated protein species relating to fiber initiation in cotton ovules. Only the differentially accumulated proteins were mapped with line drawings of the abundance changes, which are depicted in purple histograms for the wild-type and blue for the mutant. The dotted line indicates that the pathway has not yet been confirmed in cotton. Detailed descriptions are given in the text. Abbreviations: PPP, pentose phosphate pathway.

As the starting point of cotton fiber development, fiber initiation is an important process with great biological and economical values. By combining our comparative proteomic analyses results and biochemical experimental data, we for the first time present a possible fiber initiation-related protein network (Fig. 7) with different molecular events that are closely related to most of the 91 identified DAPs. These molecular events include active carbohydrate/energy metabolism to provide blocks for cell expansion, the increasing biosynthesis of amino acid and proteins, and an H_2_O_2_ burst by inhibiting the activity of enzymes in the ascorbate-glutathione cycle.

Carbohydrate/energy metabolism is the principal process providing energy and intermediates for lint fiber cell initiation and cell wall synthesis. Our proteomic results indicate that 26 identified DAPs in cotton ovules were associated with carbohydrate/energy metabolism (S1 Table). Most of these enzymes, which participate in several important metabolic pathways, such as glycolysis and the pentose-phosphate pathway and other types of carbohydrate metabolism, accumulated in the wild-type ovules but not in the *fl* mutant ovules (Fig. 7 and S2 Table), suggesting that these protein species may play critical roles in fiber initiation. Of these proteins, sucrose synthase (w58) was reported to catalyze the conversion of sucrose and nucleoside diphosphate into the corresponding nucleoside diphosphate glucose and fructose, which are closely related to cellulose synthesis and cell wall formation. Glucose-6-phosphate dehydrogenase (w10) is a rate-limiting enzyme of the pentose phosphate pathway, which is the main pathway for generating NADPH and reducing power to ensure a constant supply of NAD+ that is reduced to NADH during glycolysis. An enzymatic assay of soluble carbohydrates revealed that the *fl* mutant ovules were deficient in sugars, further reflecting the importance of carbohydrate/energy metabolism in the fiber initiation process.

The biosynthesis of biological macromolecules, especially proteins, is an important event for cell growth. Many protein biosynthesis-, folding- and assembly-associated proteins, including translation elongation factor (w34), ribosomal protein S12 (w25) and heat shock protein 70 (w1) were all preferentially accumulated in the wild-type ovules. In addition, the abundance of some amino synthesis-related proteins, such as ketol-acid reductoisomerase (w17), chorismate synthase (w9) and argininosuccinate synthase (w24), also increased in the wild-type ovules. Furthermore, the enzymes (adenosine kinase, s-adenosylmethionine synthetase, and 24-sterol C-methyltransferase) that are involved in the esterification of cell wall pectin, which is important for cell wall expansion, also accumulated in the wild-type ovules (Fig. 7).

The H_2_O_2_ level peaked at 0 dpa, when the fiber cells protruded from the ovular surface in the wild-type ovules (Fig. 6-A). This so-called H_2_O_2_ burst was not only absent in the *fl* mutant ovules but also can be inhibited by blocking the fertilization process (Fig. 6-A). Furthermore, the *in vivo* addition of H_2_O_2_ promoted fiber initiation (Fig. 6-B, C and D). These observations strongly indicate that an H_2_O_2_ burst on the day of anthesis is an important signal for cotton fiber initiation. How did this H_2_O_2_ burst form in wild-type ovules? Our comparative proteomic analyses provide a possible answer to this question. The enzymes that are involved in ROS elimination, such as sAPx and DHAR, were differently accumulated in the wild-type and *fl* mutant ovules (S1 Table and Fig. 5). An enzyme assay further revealed that the activity of sAPx and DHAR was lower in the wild-type ovules (Figs. 5 and 7), ensuring the H_2_O_2_ was not eliminated on the day of anthesis.

Through bioinformatics prediction combined with mass spectrometry identification, Zhang et al. found that 40 of the 235 differentially expressed proteins that were revealed by 2-DE-based comparative proteomics analyses of cotton fiber elongation process were phosphoproteins, suggesting that reversible phosphorylation plays an important role in fiber elongation [24]. Recently, Ma et al. used a mass spectrometry-based phosphoproteomics method to explore the phosphoproteome differences between the Xu-142 wild-type and Xu-142-*fl* mutant at -3 and 0 dpa. The results indicate that 69 of the 619 identified phosphoproteins were differentially expressed between the wild-type and *fl* mutant, and functional ontology further revealed that these proteins are mainly involved in signal transduction, protein modification, carbohydrate metabolic processes, and cell cycle and cell proliferation [43]. These studies noted that the characterization of post-translational modifications, especially phosphorylation, could greatly improve our understanding of cotton fiber development on a proteomic scale. Using a similar phosphoprotein identification method, we could further explore the phosphorylation-level regulation of the 91 DAPs to expand the protein network underlying cotton fiber initiation to a dynamic regulation network.

In summary, the dynamic changes in abundance of these 91 DAPs may indicate that the epidermal cells of the wild-type ovules could sense the initiation signal by modulating the amount of the proteins and the corresponding molecular events, eventually transforming epidermal cells into a new shape: cotton fibers. The protein network that was depicted based on our proteomics analyses could help us to systemically understand the molecular mechanism of fiber initiation, whereas further post-translational modification exploration and the in-depth functional characterization of these DAPs based on the protein network would reveal the possible key regulating proteins driving fiber initiation.

## Conclusion

Fiber cell initiation is a dynamic process that is accompanied by major protein changes at different initiation stages. In this study, lint fiber initiation-related proteins were investigated in cotton ovules at the proteomic level between wild-type cotton (*G*. *hirsutum* cv. Xu-142) and its *fuzzless-lintless* mutant. As a result, a total of 91 differentially accumulated protein species were identified through MS analysis, among which 58 were preferentially accumulated in the wild-type, and the others were preferentially accumulated in the *fl* mutant ovules. Based on the putative functions and abundance variance of the 91 identified protein species, in association with the biochemical analyses, we proposed a protein network for fiber initiation, including a series of molecular events occurring in cotton ovules (Fig. 7). The network covers many metabolic processes that mainly include redox homeostasis with H_2_O_2_ generation, carbohydrate metabolism/energy production, amino acid/protein synthesis, and the biosynthesis of cell wall components. In brief, our study presents an important resource of data integration of the molecular events occurring in the fiber initiation process. Further hypothesis-driven molecular analyses can be conducted to fully elucidate the molecular mechanisms behind fiber cell initiation.

## Materials and Methods

### Plant materials

Upland cotton *Gossypium hirsutum* L. cv. Xuzhou-142 (Xu-142) and its *fl* mutant were grown in greenhouse at 28°C/20°C with a photoperiod of 16 h light/8 h dark and a relative humidity of 60%. Cotton bolls of -3, -1, 0, +1, +3 dpa were dissected using a laminar scalpel to obtain intact ovules. The obtained ovules were immediately frozen in liquid nitrogen and then stored at -80°C until use for protein and RNA extractions. For fertilization blocking treatment, androecium together with stigma and style were removed from flowers on the evening of -1 dpa, and then the unfertilized ovules were collected at 0, +1 and +3 dpa for comparative analysis with those fertilized.

### Protein preparation and two-dimensional electrophoresis

The total proteins of the ovules were extracted using a modified phenol method as previously described [10]. The final protein pellets were dissolved in 2-DE rehydration buffer (7 M urea, 2 M thiourea, 4% CHAPS, 1% IPG buffer, and 40 mM dithiothreitol). By centrifuging at 12,000 g for 15 min, the insoluble fractions were removed. The protein concentration was determined with a BCA Protein Assay kit (Promega, Madison, USA).

Two-dimensional electrophoresis (2-DE) was performed as previously described [10, 13]. Briefly, 800 (g of proteins in 450 (l were loaded onto precast nonlinear IPG strips (24 cm, pH 3–10, GE Healthcare, Piscataway, USA), after which IEF was performed under the following conditions: 100 V for 40 min, 500 V for 40 min, 1000 V for 1 h, 4000 V for 2 h, and 8000 V for up to 80,000 Vh. SDS-PAGE was performed using 12.5% polyacrylamide gels without a stacking gel in the Ettan DALT System (GE Healthcare). Protein spots in the 2-DE gels were detected by a modified colloidal CBB G-250 staining method with blue silver [22]. Images of the gels were scanned by a UMAX PowerLook 2100XL scanner (UMAX Systems GmbH, Willich, Germany). At least triplicate protein samples were prepared using different plant materials for each 2-DE.

### 2-DE image analysis and protein identification by MS or MS/MS

The image analysis was processed with ImageMaster 2D Platinum software (version 6.0, GE Healthcare). The detection and identification of the protein spots were carried out using an automatic analysis mode followed by visual re-evaluation. The detected protein spots were quantified using the percent volume criterion. The relative volume of each spot was assumed to represent its expression level. A criterion of *p-value*<0.05 was used to define the significant difference when analyzing the parallel spots between groups with a one-way analysis of variance and Student-Newman-Keuls test using the SAS software package version 8.2 (SAS Institute).

Protein spots showing significant changes in abundance were excised from gels and in-gel digested using trypsin as previously described [10, 44]. The resulting peptide mixtures were dissolved in 0.7 μl of an α-cyano-4-hydroxycinnamic acid (CHCA) matrix solution and spotted onto a freshly cleaned target plate. After air drying, the crystallized spots were processed with a 4800 MALDI TOF/TOF Analyzer (Applied Biosystems, Framingham, USA) [12, 13]. The obtained peak lists were searched by the MASCOT software (Matrix Science, London, UK) against a *Gossypium* peptide sequence database that was created from the *Gossypium* EST database (including 507,959 EST sequences and 62,267,048 residues) [12, 13]. The samples that were not identified by peptide mass fingerprinting (PMF) were automatically submitted to MS/MS analysis with the 10 strongest precursors being selected from one MS scan. The combined MS and MS/MS information were searched by MASCOT as mentioned above. In all of the protein identification processes by either PMF or MS/MS, the successful hints were defined with a significant probability at *p*-*value*<0.05.

### Subcellular localization analysis of transiently expressed fusion proteins

Total RNA was extracted from the cotton ovules using the PureLink Plant RNA Reagent kit (Invitrogen, Carlsbad, USA) and reverse-transcribed to cDNA using TaKaRa RNA PCR Kit (TaKaRa, Dalian, China) according to the protocols provided by the manufacturers. To construct a GhsAPx-signal peptide:GFP fusion protein for localization analyses in cotton leaves, the coding sequence of the GhsAPx signal peptide was amplified with the primers APxS-F (5′-ATCCATGG *CT*ATGGCTTCCTCTCTCAACAG-3′) and APxS-R (5′-GTGGATCCGGCTACAGAGCTAAACCCTC-3′). To facilitate vector construction, *Nco*I and *Bam*HI sites (underlined) were introduced into the 5′-end sites of the two primers, respectively, and two nucleotides (CT, italic) were added to the forward primer adjacent to the *Nco*I site to prevent frame shifting. The PCR product was then cloned into the *Nco*I-*Bam*HI site of the pCK-GFP plasmid (a gift from A.M. Duchene, BMP, Strasbourg, France) to generate a fusion in frame with the coding sequence for enhanced GFP, in which expression is driven by the CaMV 35S promoter. The fusion construct or control GFP vector was then introduced into the cotton leaf by particle bombardment using a Bio-Rad Biolistic PDS 1000/He system. Plasmid DNA (1 mg) was used to coat 0.5 mg of 1-μm gold particles. The cotton leaf had previously been incubated on MS agar plates in the dark at 22°C for 24 h. The initial pressure of the bombardment was 1100 psi, and the traveling distance of the particles to the plant tissues was 6 cm. The bombarded tissues were placed on the same agar plates and incubated at 22°C for 24 h in the dark, followed by the monitoring of the localization of GFP and the autofluorescence of the chloroplast with a FluoView FV300 confocal microscope (Olympus Corporation, Tokyo, Japan).

### Amino acid sequencing

The N-terminal amino acid sequencing of the four GhsAPx protein spots was performed by automated Edman degradation. Phenylthiohydantoin derivatives were detected using a pulse liquid automatic sequencer (Applied Biosystems, Fullerton, USA). The resulting sequences were analyzed using the protein analysis system (ExPASy) server of the Swiss Institute of Bioinformatics (SIB, Lausanne, Switzerland).

### Scanning electron microscopy (SEM)

Cotton ovules were collected at the fiber initiation stages of -3, -1, 0, +1, and +3 dpa as described above. Scanning electron microscopy of the cotton ovules was performed according to Craig and Beaton [45]. The specimens were viewed and photographed with a Hitachi-SEM-S300 (Hitachi, Tokyo, Japan).

### Determination of the carbohydrate contents

The contents of glucose, fructose, sucrose and starch in cotton ovules were determined enzymatically with an F-kit (Roche, Mannheim, Germany) on a spectrophotometer at 340 nm as previously described [46].

### Enzyme assay

The enzyme activities of APx isoforms in different subcellular locations were assayed as described by Amako et al. [47] with some modifications. Briefly, the cotton ovules were ground to a fine powder in liquid nitrogen and then resuspended in 10 mM potassium phosphate buffer (pH 7.0) containing 1 mM ascorbate, 20% (w/v) sorbitol, 1 mM EDTA, and 0.1% (w/v) phenylmethanesulfonyl fluoride. The homogenate was squeezed through four layers of cheesecloth and then centrifuged at 12,000 g for 30 min. The obtained soluble fraction conserved the activities of the sAPX and cAPX isozymes. A 5-(l aliquot of the supernatant was added to 2.0 ml of N_2_-bubbling 50 mM potassium phosphate buffer (pH 7.0) containing 10 (M H_2_O_2_. At 3, 4, 5, and 6 min after the start of the incubation, the incubated mixture (1.98 ml) was sampled and mixed with 10 (l of 100 mM ascorbate to terminate the inactivation. The residual activity of the APxs was then assayed by adding 10 (l of 20 (M H_2_O_2_. The oxidation of ascorbate was followed by a decrease in the A_290_, and the results are plotted on the graph. The ratio of the cAPx and sAPx activities was calculated from the inactivation curve of each isozyme. The pellet from centrifugation at 12,000 g was washed and resuspended in 10 mM potassium phosphate buffer (pH 7.0) containing 1 mM ascorbate. The tAPx and mAPx proteins were conserved in this fraction, and their total activities were assayed by recording the speed of the decrease of A_290_. The protein content of each fraction was quantified using the Bradford method [48].

DHAR activity in the same supernatant after centrifugation at 12,000 g was assayed as previously described in an assay mixture containing 50 mM potassium phosphate buffer (pH 7.8), 0.1 mM dehydroascorbate (Sigma-Aldrich, St Louis, USA) and 2 mM reductive glutathione (Sigma-Aldrich) by monitoring the absorbance change at 265 nm [49].

### Determination of H_2_O_2_, ascorbate and dehydroascorbate

H_2_O_2_ was measured as described by Okuda [50]. The ovules were ground using a pestle and mortar in liquid nitrogen. The sample powder was suspended in 1 ml of 3% (v/v) HClO_4_ containing 2.5 mM EDTA and centrifuged at 20,000 g for 5 min at 4°C. The supernatant was neutralized with 1 M KOH to pH 7.5 and centrifuged at 20,000 g for 5 min at 4°C. The obtained supernatant was filtered through an anion-exchange column. A total of 400 (l of 12.5 mM DMAB and 80 (l of MBTH were added to 1 ml of the column elutes. The reaction was started by the addition of 20 (l of peroxidase (0.25 units) at 25°C. The H_2_O_2_ in the sample was determined by monitoring the increased absorbance at 590 nm with an Ultrospec 3300 Spectrophotometer (GE Healthcare).

Ascorbate and dehydroascorbate were measured as previously described [51]. The ovules powder was suspended in 1 ml of 6% (v/v) HClO_4_ and centrifuged at 20,000 g for 10 min at 4°C. A 100-(l aliquot of ovule extract was added directly to 900 (l of 200 mM succinate buffer (pH 12.7). The A_265_ was immediately recorded and again 5 min after the addition of 5 units of ascorbate oxidase. To determine the total ascorbate, the ovule extract was adjusted to pH 6.0 with 1.25 M K_2_CO_3_ and centrifuged at 20,000 g for 5 min. The supernatant was incubated with 10 mM dithiothreitol in 50 mM HEPES-KOH buffer (pH 7.5) for 10 min at 25°C. Then, 100 (l of the mixture solution was directly added to 900 (l of 200 mM succinate buffer (pH 6.0). The resultant solution was assayed as described above. The difference between the total ascorbate and ascorbate contents was considered the content of dehydroascorbate. Finally, the ratio of ascorbate/dehydroascorbate was calculated.

## Supporting Information

S1 Fig Representative PMF and MS/MS identification results. (TIFF)

S1 Table Differentially displayed proteins identified by PMF or MS/MS. (DOCX)

S2 Table Protein abundance of differentially displayed proteins. (DOCX)
