# Supplementary figures and images for: Comparative Proteomic and Biochemical Analyses Reveal Different Molecular Events Occurring in the Process of Fiber Initiation between Wild-Type Allotetraploid Cotton and Its Fuzzless-Lintless Mutant
Source: PLoS One. 2015 Feb 20;10(2):e0117049. doi: 10.1371/journal.pone.0117049 (PMC4336136; doi:10.1371/journal.pone.0117049)

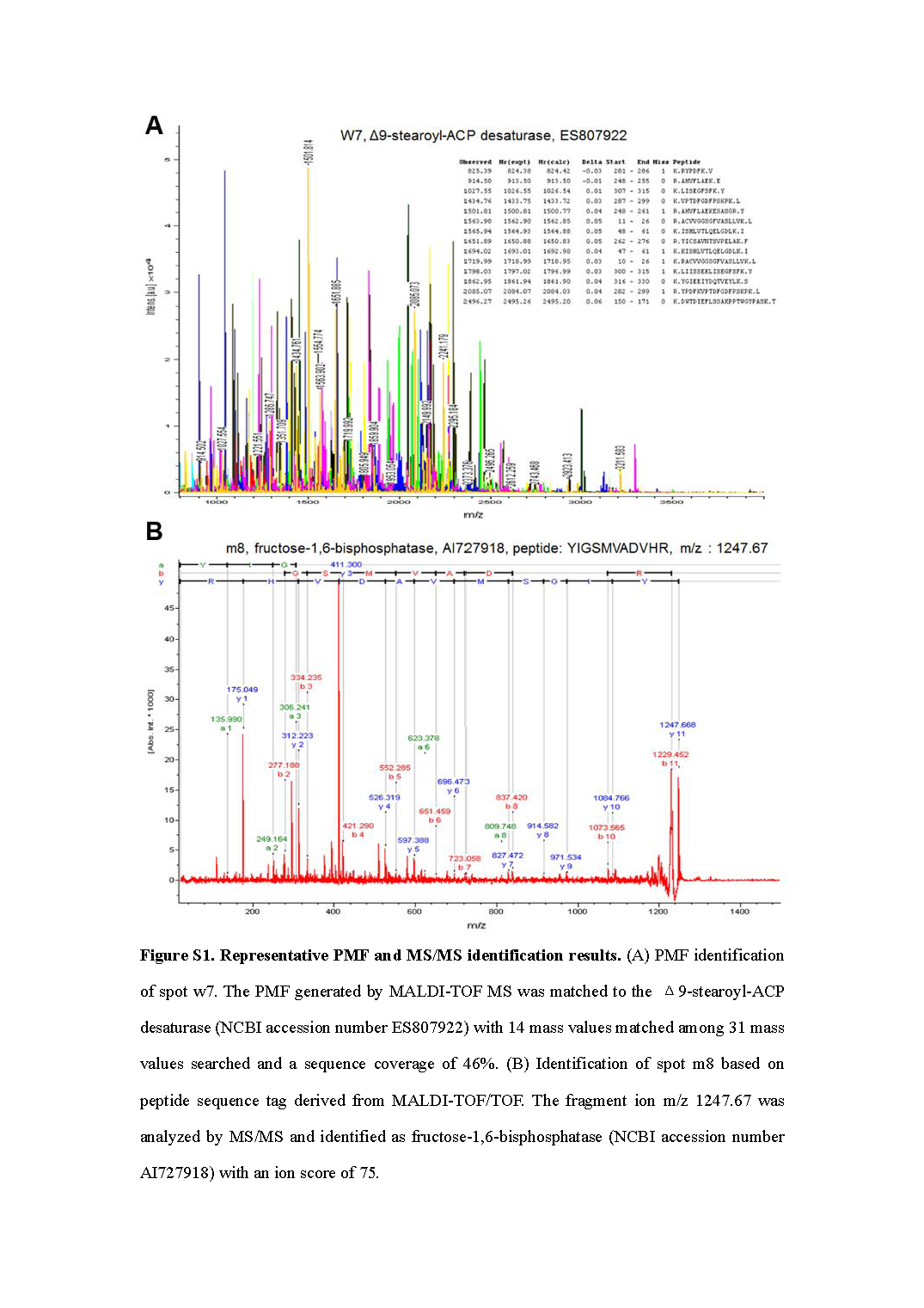

Supplement: S1 Fig — (TIFF) [file pone.0117049.s001.tiff]
