# Supplementary material for: Comparative Proteomic and Biochemical Analyses Reveal Different Molecular Events Occurring in the Process of Fiber Initiation between Wild-Type Allotetraploid Cotton and Its Fuzzless-Lintless Mutant
Source: PLoS One. 2015 Feb 20;10(2):e0117049. doi: 10.1371/journal.pone.0117049 (PMC4336136; doi:10.1371/journal.pone.0117049)
Supplement: S1 Table — (DOCX) [file pone.0117049.s002.docx]

**Table S1. Differentially displayed proteins identified by PMF or MS/MS**

| **Spot ID** | **Protein name** | **NCBI acc. No.** | **Species** | **Mascot score** | **M^a^** | **C^b^ (%)** | **MW(kDa) pre/obs** | **p*I* pre/obs** | **Stages (DPA)** | **FC^c^** |
| --- | --- | --- | --- | --- | --- | --- | --- | --- | --- | --- |
| **Energy/Carbohydrate Metabolism** | | | | | | | | | | |
| w8 | isocitrate dehydrogenase | CO101929 | *Gossypium raimondii* | 68 | 13 | 45 | 39.76/38.77 | 7.47/6.74 | -3,-1,0,+1,+3 | 14.76 |
| w10 | glucose-6-phosphate dehydrogenase | BQ414189 | *Gossypium hirsutum* | 97 | 1* | 7 | 58.77/55.13 | 7.05/8.52 | -3,-1,0,+1,+3 | 26.29 |
| w11 | putative transketolase | ES819000 | *Gossypium hirsutum* | 162 | 2* | 12 | 80.51/70.78 | 6.80/6.14 | -3,-1,0,+1,+3 | 2.46 |
| w12 | cytoplasmic aconitate hydratase/ citrate hydro-lyase/aconitase | CO071376 | *Gossypium raimondii* | 83 | 11 | 48 | 108.18/95.64 | 7.18/5.97 | -3,-1,0,+1,+3 | 8.86 |
| w15 | succinyl-CoA ligase β-chain | DT572135 | *Gossypium hirsutum* | 109 | 21 | 52 | 45.28/40.43 | 6.77/5.23 | -3,-1,0,+1,+3 | 2.14 |
| w16 | triosephosphate isomerase | CO082348 | *Gossypium raimondii* | 134 | 18 | 56 | 33.11/29.08 | 7.14/5.94 | -3,-1,0,+1,+3 | 12.19 |
| w29 | acyltransferase-like protein | DT550542 | *Gossypium hirsutum* | 94 | 13 | 54 | 49.95/50.15 | 6.71/5.87 | +1,+3 | 15.53 |
| w31 | acyltransferase-like protein | ES829997 | *Gossypium hirsutum* | 173 | 4* | 22 | 49.95/50.15 | 6.71/5.87 | +1,+3 | 2.88 |
| w50 | acyltransferase-like protein | ES847325 | *Gossypium hirsutum* | 180 | 2* | 14 | 49.95/55.44 | 6.71/5.61 | +3 | 9.52 |
| w32 | phosphoglycerate kinase | DT564550 | *Gossypium hirsutum* | 85 | 12 | 46 | 42.26/38.66 | 6.29/6.45 | +1,+3 | 10.44 |
| w35 | carboxylesterase | DT545462 | *Gossypium hirsutum* | 121 | 20 | 50 | 30.06/32.78 | 5.98/5.68 | +1,+3 | 2.42 |
| w36 | glutathione S-transferase | ES846286 | *Gossypium hirsutum* | 140 | 22 | 66 | 31.80/30.78 | 8.36/5.70 | +1,+3 | 1.54 |
| w41 | anthranilate N-benzoyl transferase protein | ES822972 | *Gossypium arboreum* | 223 | 3* | 16 | 48.71/40.76 | 6.95/6.71 | +3 | 1.78 |
| w52 | reversibly glycosylated polypeptide | CO130114 | *Gossypium raimondii* | 114 | 17 | 70 | 40.84/39.10 | 6.36/5.88 | +3 | 1.69 |
| w55 | UDP-D-glucose dehydrogenase | DT570678 | *Gossypium hirsutum* | 100 | 14 | 59 | 52.81/52.20 | 6.62/6.27 | +3 | 1.61 |
| w58 | sucrose synthase 1 | ES808669 | *Gossypium hirsutum* | 72 | 13 | 44 | 92.54/89.60 | 6.58/6.15 | +3 | 3.30 |
| m4 | vacuolar H^+^-ATPase subunit E | CO092517 | *Gossypium raimondii* | 139 | 21 | 48 | 27.16/31.72 | 7.01/6.93 | -3,-1,0,+1,+3 | 0.21 |
| m5 | NADH-cytochrome B5 reductase | ES845779 | *Gossypium raimondii* | 155 | 19 | 70 | 35.75/29.60 | 9.17/7.18 | -3,-1,0,+1,+3 | 0.06 |
| m8 | fructose-1,6-bisphosphatase | AI727918 | *Gossypium hirsutum* | 75 | 2* | 10 | 37.23/38.78 | 6.50/5.88 | -3,-1,0,+1,+3 | 0.06 |
| m9 | transketolase | DW232053 | *Gossypium hirsutum* | 86 | 13 | 58 | 80.51/72.46 | 6.80/6.09 | -3,-1,0,+1,+3 | 0.14 |
| m11 | class I endochitinase | ES798040 | *Gossypium hirsutum* | 106 | 15 | 59 | 34.66/31.32 | 6.67/5.37 | -3,-1,0,+1,+3 | 0.24 |
| m15 | β-aldehyde dehydrogenase | CO071599 | *Gossypium raimondii* | 95 | 15 | 68 | 54.71/64.34 | 5.45/5.46 | 0,+1,+3 | 0.50 |
| m24 | thiazole biosynthetic enzyme | DT553222 | *Gossypium hirsutum* | 96 | 15 | 66 | 37.98/34.11 | 5.86/4.82 | +1,+3 | 0.62 |
| m28 | 2-nitropropane dioxygenase | DT551724 | *Gossypium hirsutum* | 121 | 16 | 67 | 36.01/38.22 | 5.23/5.19 | +3 | 0.21 |
| **Redox Homeostasis** | | | | | | | | | | |
| w2 | lactoylglutathione lyase/ glyoxalase I | CO076010 | *Gossypium raimondii* | 120 | 16 | 77 | 32.51/34.57 | 5.77/5.76 | -3,-1,0,+1,+3 | 23.28 |
| m13 | lactoylglutathione lyase/ glyoxalase I | CO076010 | *Gossypium raimondii* | 117 | 17 | 65 | 32.51/34.85 | 5.77/5.58 | -3,-1,0,+1,+3 | 0.54 |
| w3 | chloroplast stromal ascorbate peroxidase | DT545161 | *Gossypium hirsutum* | 203 | 21 | 78 | 37.29/32.88 | 8.97/5.81 | -3,-1,0,+1,+3 | 18.35 |
| w14 | chloroplast stromal ascorbate peroxidase | DT545161 | *Gossypium hirsutum* | 121 | 15 | 62 | 37.29/32.33 | 8.97/5.93 | -3,-1,0,+1,+3 | 4.68 |
| m7 | chloroplast stromal ascorbate peroxidase | DT545161 | *Gossypium hirsutum* | 153 | 16 | 79 | 37.29/32.33 | 8.97/5.93 | -3,-1,0,+1,+3 | 0.25 |
| m21 | chloroplast stromal ascorbate peroxidase | DT545161 | *Gossypium hirsutum* | 187 | 20 | 89 | 37.29/31.61 | 8.97/5.98 | +1,+3 | 0.37 |
| w4 | aldo-keto reductase | BG442284 | *Gossypium arboreum* | 76 | 10 | 63 | 34.99/33.81 | 7.94/5.82 | -3,-1,0,+1,+3 | 10.18 |
| w18 | dehydroascorbate reductase | ES815517 | *Gossypium hirsutum* | 112 | 14 | 56 | 23.54/25.41 | 6.63/7.12 | -3,-1,0,+1,+3 | 10.38 |
| m3 | dehydroascorbate reductase | ES815517 | *Gossypium hirsutum* | 138 | 16 | 75 | 23.54/24.96 | 6.63/6.17 | -3,-1,0,+1,+3 | 0.11 |
| w27 | benzoquinone reductase 2 | ES849598 | *Gossypium hirsutum* | 82 | 15 | 55 | 21.66/26.14 | 6.53/5.91 | +1,+3 | 6.70 |
| w53 | benzoquinone reductase 2 | DW481092 | *Gossypium hirsutum* | 473 | 6* | 33 | 21.66/26.05 | 6.53/5.71 | +3 | 2.01 |
| w28 | benzoquinone reductase 1 | ES805177 | *Gossypium hirsutum* | 670 | 7* | 44 | 21.75/25.14 | 7.85/6.16 | +1,+3 | 6.30 |
| w30 | polyphenol oxidase | DT555541 | *Gossypium hirsutum* | 83 | 3* | 13 | 65.76/74.76 | 8.24/5.63 | +1,+3 | 11.68 |
| w39 | polyphenol oxidase, chloroplast precursor | ES841032 | *Gossypium hirsutum* | 114 | 18 | 76 | 66.20/72.74 | 6.97/5.83 | +3 | 14.83 |
| **Amino Acid Biosynthesis** | | | | | | | | | | |
| w6 | adenosine kinase | ES809736 | *Gossypium hirsutum* | 90 | 13 | 67 | 37.37/26.90 | 5.30/6.57 | -3,-1,0,+1,+3 | 10.92 |
| w9 | chorismate synthase, chloroplast precursor | ES829368 | *Gossypium hirsutum* | 87 | 1* | 5 | 46.47/40.69 | 8.65/6.82 | -3,-1,0,+1,+3 | 8.18 |
| w24 | argininosuccinate synthase | DW008598 | *Gossypium hirsutum* | 77 | 11 | 72 | 58.84/20.61 | 5.34/5.47 | +1,+3 | 8.98 |
| w40 | diaminopimelate decarboxylase | DW479576 | *Gossypium hirsutum* | 98 | 15 | 58 | 54.06/44.76 | 7.05/6.00 | +3 | 7.46 |
| w17 | ketol-acid reductoisomerase, chloroplast precursor | DW518448 | *Gossypium hirsutum* | 111 | 17 | 51 | 63.80/58.26 | 6.99/5.74 | -3,-1,0,+1,+3 | 12.68 |
| m12 | ketol-acid reductoisomerase, chloroplast precursor | DW518448 | *Gossypium hirsutum* | 81 | 15 | 49 | 63.80/53.13 | 6.99/5.79 | -3,-1,0,+1,+3 | 0.22 |
| w46 | 5-methyltetrahydropteroyltriglutamate/homocysteine methyltransferase | DT545711 | *Gossypium hirsutum* | 78 | 13 | 45 | 84.66/81.80 | 6.47/6.27 | +3 | 1.63 |
| w56 | 5-methyltetrahydropteroyltriglutamate/homocysteine methyltransferase | EV484021 | *Gossypium hirsutum* | 114 | 15 | 65 | 84.66/81.80 | 6.47/6.10 | +3 | 1.81 |
| w57 | 5-methyltetrahydropteroyltriglutamate/homocysteine methyltransferase | ES813339 | *Gossypium hirsutum* | 110 | 16 | 51 | 84.66/81.80 | 6.47/6.58 | +3 | 1.55 |
| w48 | 24-sterol C-methyltransferase | DT561542 | *Gossypium hirsutum* | 76 | 12 | 42 | 38.94/35.85 | 5.69/5.69 | +3 | 13.8 |
| w51 | s-adenosylmethionine synthetase | DT544033 | *Gossypium hirsutum* | 183 | 23 | 87 | 40.09/41.70 | 5.59/5.59 | +3 | 14.59 |
| **Protein Folding and Stabilization** | | | | | | | | | | |
| w1 | heat shock protein 70 | DT544347 | *Gossypium hirsutum* | 117 | 19 | 57 | 71.20/70.78 | 4.81/5.75 | -3,-1,0,+1,+3 | 11.39 |
| w22 | 20S proteasome α-subunit /endopeptidase/threonine-type endopeptidase | CO097957 | *Gossypium raimondii* | 129 | 17 | 51 | 27.11/30.13 | 7.62/7.03 | 0,+1,+3 | 1.87 |
| m26 | serpin/putative serine protease inhibitor | ES809559 | *Gossypium hirsutum* | 103 | 14 | 80 | 42.73/40.26 | 6.91/6.64 | +3 | 0.52 |
| w43 | chaperonin containing t-complex protein 1, γ-subunit | DT568010 | *Gossypium hirsutum* | 116 | 15 | 78 | 60.38/26.05 | 6.53/5.99 | +3 | 12.83 |
| m31 | chaperonin containing t-complex protein 1, γ-subunit | CO094823 | *Gossypium raimondii* | 135 | 19 | 76 | 60.38/58.84 | 6.53/6.24 | +3 | 0.09 |
| m32 | chaperonin containing t-complex protein 1, γ-subunit | CO094823 | *Gossypium raimondii* | 135 | 19 | 75 | 60.38/58.84 | 6.53/6.29 | +3 | 0.12 |
| **Protein Synthesis** | | | | | | | | | | |
| w25 | ribosomal protein S12 | DW476179 | *Gossypium hirsutum* | 65 | 6 | 44 | 14.99/18.90 | 5.59/5.90 | +1,+3 | 2.01 |
| w34 | eukaryotic translation elongation factor | DW227914 | *Gossypium hirsutum* | 102 | 16 | 67 | 90.03/32.60 | 6.06/5.53 | +1,+3 | 3.54 |
| m2 | 40S ribosomal protein SA | DT563374 | *Gossypium hirsutum* | 131 | 17 | 68 | 33.85/41.50 | 4.76/4.67 | -3,-1,0,+1,+3 | 0.03 |
| m17 | basic transcription factor 3 | ES801107 | *Gossypium hirsutum* | 100 | 14 | 80 | 17.62/22.03 | 7.54/5.61 | +1,+3 | 0.45 |
| m25 | pre-mRNA-splicing factor | DT561343 | *Gossypium hirsutum* | 122 | 3* | 14 | 57.56/55.69 | 6.50/5.93 | +1,+3 | 0.25 |
| **Fatty Acid Biosynthesis** | | | | | | | | | | |
| w7 | Δ^9^-stearoyl-ACP desaturase | ES807922 | *Gossypium hirsutum* | 73 | 14 | 46 | 45.23/40.34 | 6.36/5.53 | -3,-1,0,+1,+3 | 31.94 |
| m22 | plastidic pyruvate kinase β-subunit 1 | DR457233 | *Gossypium hirsutum* | 72 | 11 | 80 | 63.57/58.86 | 7.06/5.81 | +1,+3 | 0.54 |
| m27 | hydroxyacyl-ACP dehydrase | EV496046 | *Gossypium hirsutum* | 153 | 4* | 18 | 24.16/20.70 | 6.59/5.64 | +3 | 0.09 |
| m29 | enoyl-ACP reductase | ES813690 | *Gossypium hirsutum* | 86 | 12 | 59 | 41.21/38.38 | 6.36/5.01 | +3 | 0.08 |
| **Nucleotide metabolism** | | | | | | | | | | |
| w44 | cytidylate kinase/uridylate kinase | ES841989 | *Gossypium hirsutum* | 75 | 11 | 52 | 23.29/26.05 | 6.16/5.83 | +3 | 23.94 |
| w47 | adenosine kinase | DW479318 | *Gossypium hirsutum* | 124 | 18 | 73 | 37.38/40.32 | 5.03/5.49 | +3 | 1.70 |
| m16 | deoxyuridine 5’-triphosphate nucleotidohydrolase family | DW499184 | *Gossypium hirsutum* | 99 | 12 | 77 | 19.48/22.48 | 6.11/5.58 | +1,+3 | 0.05 |
| **Anthocyanidin Metabolism** | | | | | | | | | | |
| w54 | flavonoid 3-glucosyltransferase | DT566679 | *Gossypium hirsutum* | 502 | 5* | 26 | 49.36/43.00 | 6.57/6.19 | +3 | 1.64 |
| m10 | anthocyanidin reductase 2 | ES796849 | *Gossypium hirsutum* | 108 | 17 | 51 | 36.30/40.99 | 5.25/5.48 | -3,-1,0,+1,+3 | 0.68 |
| m23 | anthocyanidin reductase 2 | ES815227 | *Gossypium hirsutum* | 130 | 20 | 46 | 36.30/40.43 | 5.25/5.49 | +1,+3 | 0.45 |
| **Cytoskeleton** | | | | | | | | | | |
| w13 | actin13 | CO088772 | *Gossypium raimondii* | 93 | 13 | 67 | 41.67/43.87 | 5.16/5.80 | -3,-1,0,+1,+3 | 4.02 |
| w37 | actin 2 | DT546638 | *Gossypium hirsutum* | 124 | 16 | 67 | 41.73/39.98 | 5.30/5.75 | +1,+3 | 9.92 |
| w33 | annexin | ES843548 | *Gossypium hirsutum* | 270 | 29 | 79 | 35.83/33.43 | 7.25/6.95 | +1,+3 | 2.35 |
| w19 | annexin 1 | ES805410 | *Gossypium hirsutum* | 192 | 26 | 72 | 36.05/32.51 | 6.62/6.23 | -3,-1,0,+1,+3 | 1.55 |
| m6 | annexin 1 | CO079002 | *Gossypium raimondii* | 180 | 23 | 63 | 36.05/33.23 | 6.62/6.28 | -3,-1,0,+1,+3 | 0.05 |
| **Defense Responses** | | | | | | | | | | |
| w23 | major latex-like protein | ES826363 | *Gossypium hirsutum* | 107 | 12 | 71 | 17.11/14.68 | 5.34/5.47 | 0,+1,+3 | 3.10 |
| m14 | major latex-like protein | DN827285 | *Gossypium hirsutum* | 278 | 3* | 24 | 17.93/17.25 | 6.26/5.60 | -1,0,+1,+3 | 0.11 |
| w49 | DNA-damage-repair/toleration protein DRT102 | DT553723 | *Gossypium hirsutum* | 154 | 19 | 67 | 33.43/35.16 | 4.97/5.20 | +3 | 9.97 |
| m20 | 21 kDa trypsin inhibitor | DW501024 | *Gossypium hirsutum* | 167 | 2* | 18 | 24.12/20.44 | 6.59/5.87 | +1,+3 | 0.42 |
| m33 | universal stress protein family protein | DT462063 | *Gossypium hirsutum* | 73 | 12 | 49 | 19.92/22.17 | 6.24/5.79 | +3 | 0.11 |
| **Signaling Transduction** | | | | | | | | | | |
| w38 | phosphatidylinositol 4-OH kinase β-1/1-phosphatidylinositol 4-kinase | CO114426 | *Gossypium raimondii* | 65 | 9 | 51 | 126.35/24.09 | 5.34/5.62 | +1,+3 | 7.02 |
| m30 | ACC oxidase 1 | ES831910 | *Gossypium hirsutum* | 95 | 17 | 64 | 36.21/38.57 | 4.75/4.80 | +3 | 0.13 |
| **Nucleocytoplasmic Transport** | | | | | | | | | | |
| w5 | glycine-rich RNA-binding protein | CO093044 | *Gossypium raimondii* | 84 | 11 | 78 | 17.03/14.91 | 8.41/7.06 | -3,-1,0,+1,+3 | 12.78 |
| m19 | glycine-rich protein | DW520334 | *Gossypium hirsutum* | 74 | 10 | 66 | 17.64/17.07 | 6.50/5.54 | +1,+3 | 0.16 |
| **Unclassified Proteins** | | | | | | | | | | |
| w20 | conserved hypothetical protein | DW490590 | *Gossypium hirsutum* | 106 | 2* | 18 | 18.88/98.85 | 5.81/5.97 | 0,+1,+3 | 3.67 |
| w21 | conserved hypothetical protein | DW490590 | *Gossypium hirsutum* | 82 | 13 | 60 | 18.88/20.97 | 5.81/5.69 | 0,+1,+3 | 14.47 |
| w26 | conserved hypothetical protein | ES847094 | *Gossypium hirsutum* | 103 | 15 | 74 | 18.88/20.68 | 5.81/5.95 | +1,+3 | 4.34 |
| w42 | conserved hypothetical protein | ES847094 | *Gossypium hirsutum* | 223 | 15 | 69 | 18.88/19.49 | 5.81/6.78 | +3 | 8.00 |
| w45 | conserved hypothetical protein | DW518109 | *Gossypium hirsutum* | 578 | 7* | 56 | 18.88/17.92 | 5.81/5.67 | +3 | 14.28 |
| m1 | unknown protein | CO105642 | *Gossypium raimondii* | 61 | 2* | 17 | 74.67/68.52 | 5.63/6.02 | -3,-1,0,+1,+3 | 0.06 |
| m18 | hypothetical protein | BM360015 | *Gossypium arboreum* | 62 | 1* | 11 | 20.77/31.49 | 5.92/5.54 | +1,+3 | 0.59 |

Abbreviations:

^a^ Number of mass value matched peptides, the number marked with * indicates the peptide fragment(s) sequenced by MS/MS.

^b^ Sequence coverage.

^c^ The max fold change of relative abundance of specific spot at the five stages between wt and *fl* mutant (higher- versus lower-abundance spot). Detailed information is listed in Supporting Information Table S2.

.
