# Supplementary material for: Comparative Proteomic and Biochemical Analyses Reveal Different Molecular Events Occurring in the Process of Fiber Initiation between Wild-Type Allotetraploid Cotton and Its Fuzzless-Lintless Mutant
Source: PLoS One. 2015 Feb 20;10(2):e0117049. doi: 10.1371/journal.pone.0117049 (PMC4336136; doi:10.1371/journal.pone.0117049)
Supplement: S2 Table — (DOCX) [file pone.0117049.s003.docx]

**Table S2. Protein abundance of differentially displayed proteins**

| **Spot ID** | **Protein abundance (%volume, w/m)** | | | | |
| --- | --- | --- | --- | --- | --- |
|  | **-3 DPA** | **-1 DPA** | **0 DPA** | **+1 DPA** | **+3 DPA** |
| w1 | 0.0252**/**0.0027 | 0.0212**/**0.0022 | 0.0258**/**0.0016 | 0.0306**/**0.0016 | 0.01567**/**0.0023 |
| w2† | 0.136**/**0.0068 | 0.152**/**0.0052 | 0.175**/**0.0070 | 0.133**/**0.008 | 0.135**/**0.0044 |
| w3 | 0.0302**/**0.0016 | 0.0327**/**0.0012 | 0.0323**/**0.0015 | 0.0512**/**0.0035 | 0.0499**/**0.0029 |
| w4 | 0.0215**/**0.0023 | 0.0218**/**0.0023 | 0.0241**/**0.0011 | 0.0184**/**0.0017 | 0.0140**/**0.0024 |
| w5 | 0.0402**/**0.0012 | 0.0228**/**0.0015 | 0.0199**/**0.0028 | 0.0317**/**0.0034 | 0.0349**/**0.0028 |
| w6 | 0.0315**/**0.0038 | 0.0306**/**0.0032 | 0.0371**/**0.0032 | 0.0298**/**0.0021 | 0.025**/**0.0018 |
| w7† | 0.128*/*0.0044 | 0.223**/**0.0047 | 0. 165**/**0.0076 | 0.218**/**0.0059 | 0.157**/**0.0053 |
| w8† | 0.0235**/**0.0011 | 0.0244**/**0.0011 | 0.0326**/**0.0016 | 0.0320**/**0.002 | 0.0248**/**0.0035 |
| w9 | 0.0105**/**0.0012 | 0.0311**/**0.0005 | 0.0390**/**0.0012 | 0.0219**/**0.0045 | 0.0129**/**0.0067 |
| w10† | 0.028**/**0.0006 | 0.0396**/**0.0008 | 0.0505**/**0.0016 | 0.0227**/**0.0011 | 0.0117**/**0.0017 |
| w11 | 0.0206**/**0.0049 | 0.0312**/**0.0144 | 0.0337**/**0.0122 | 0.029**/**0.0138 | 0.0318**/**0.0142 |
| w12 | 0.0221**/**0.0046 | 0.0172**/**0.0011 | 0.0183**/**0.0009 | 0.0265**/**0.0022 | 0.0205**/**0.0030 |
| w13† | 0.0539**/**0.0093 | 0.0484**/**0.0019 | 0.0503**/**0.0212 | 0.0455**/**0.0118 | 0.0505**/**0.0176 |
| w14† | 0.0426**/**0.0028 | 0.0496**/**0.0013 | 0.043**/**0.0034 | 0.0481**/**0.0036 | 0.0332**/**0.0031 |
| w15 | 0.0772**/**0.0506 | 0.0932**/**0.0393 | 0.0864**/**0.0336 | 0.0777**/**0.0330 | 0.0474**/**0.0216 |
| w16 | 0.0454**/**0.0038 | 0.0789**/**0.0033 | 0.0298**/**0.0033 | 0.0379**/**0.0047 | 0.0517**/**0.0049 |
| w17† | 0.0553**/**0.0045 | 0.0622**/**0.0065 | 0.0557**/**0.0048 | 0.0713**/**0.006 | 0.0802**/**0.0038 |
| w18† | 0.0203**/**0.0018 | 0.014**/**0.0019 | 0.0164**/**0.0016 | 0.0153**/**0.0015 | 0.0181**/**0.0013 |
| w19 | 0.210**/**0.140 | 0.227**/**0.144 | 0.172**/**0.104 | 0.224**/**0.145 | 0.216**/**0.145 |
| w20 | - | - | 0.0111**/**0.0009 | 0.0142**/**0.0004 | 0.0118**/**0.0007 |
| w21 | - | - | 0.0295**/**0.0019 | 0.118**/**0.01 | 0.11**/**0.0059 |
| w22 | - | - | 0.0679**/**0.0369 | 0.0427**/**0.0242 | 0.0672**/**0.0339 |
| w23 | - | - | 0.0549**/**0.0237 | 0.0942**/**0.0248 | 0.0815**/**0.0259 |
| w24 | - | - | - | 0.0263**/**0.0025 | 0.0231**/**0.0030 |
| w25 | - | - | - | 0.0212**/**0.0141 | 0.0117**/**0.0023 |
| w26 | - | - | - | 0.0713**/**0.0135 | 0.0892**/**0.0235 |
| w27 | - | - | - | 0.0243**/**0.0055 | 0.0447**/**0.0048 |
| w28 | - | - | - | 0.0388**/**0.0085 | 0.0715**/**0.0090 |
| w29 | - | - | - | 0.137**/**0.0092 | 0.155**/**0.0096 |
| w30 | - | - | - | 0.023**/**0.0021 | 0.0202/0.0016 |
| w31 | - | - | - | 0.162**/**0.064 | 0.164**/**0.049 |
| w32 | - | - | - | 0.0231**/**0.0027 | 0.0176**/**0.0012 |
| w33 | - | - | - | 0.0903**/**0.0362 | 0.14**/**0.062 |
| w34 | - | - | - | 0.0434**/**0.0100 | 0.0610**/**0.0195 |
| w35 | - | - | - | 0.0546**/**0.0258 | 0.0367**/**0.0120 |
| w36 | - | - | - | 0.0614**/**0.0132 | 0.0696**/**0.0177 |
| w37 | - | - | - | 0.0426**/**0.0046 | 0.0616**/**0.0059 |
| w38 | - | - | - | 0.0197**/**0.0027 | 0.0203**/**0.0030 |
| w39 | - | - | - | - | 0.0445**/**0.0030 |
| w40 | - | - | - | - | 0.0403**/**0.0054 |
| w41 | - | - | - | - | 0.112**/**0.063 |
| w42 | - | - | - | - | 0.0128**/**0.0016 |
| w43 | - | - | - | - | 0.0231**/**0.0018 |
| w44 | - | - | - | - | 0.0407**/**0.0017 |
| w45 | - | -- | - | - | 0.0357**/**0.0025 |
| w46 | - | - | - | - | 0.113**/**0.0692 |
| w47 | - | - | - | - | 0.25**/**0.147 |
| w48 | - | - | - | - | 0.0138**/**0.0010 |
| w49 | - | - | - | - | 0.0289**/**0.0029 |
| w50 | - | - | - | - | 0.0314**/**0.0033 |
| w51 | - | - | - | - | 0.321**/**0.220 |
| w52 | - | - | - | - | 0.104**/**0.614 |
| w53 | - | - | - | - | 0.043**/**0.214 |
| w54 | - | - | - | - | 0.0766**/**0.0039 |
| w55 | - | - | - | - | 0.026**/**0.0161 |
| w56 | - | - | - | - | 0.124**/**0.0684 |
| w57 | - | - | - | - | 0.0920**/**0.0593 |
| w58 | - | - | - | - | 0.0179/0.0543 |
| m1‡ | 0.0005**/**0.0301 | 0.0007**/**0.0232 | 0.0014**/**0.0195 | 0.0017**/**0.0168 | 0.0016**/**0.0185 |
| m2‡ | 0.0029**/**0.0703 | 0.0029**/**0.0755 | 0.0023**/**0.0693 | 0.0031**/**0.113 | 0.0019**/**0.0808 |
| m3 | 0.0031**/**0.0209 | 0.0028**/**0.0255 | 0.0022**/**0.0275 | 0.0017**/**0.0228 | 0.0030**/**0.0190 |
| m4 | 0.0038**/**0.0453 | 0.0041**/**0.0406 | 0.0137**/**0.0280 | 0.0081**/**0.0263 | 0.0061**/**0.0316 |
| m5‡ | 0.0041**/**0.0586 | 0.0028**/**0.0445 | 0.0018**/**0.0417 | 0.0027**/**0.0292 | 0.0024**/**0.0492 |
| m6‡ | 0.0028**/**0.0990 | 0.0035**/**0.0718 | 0.0042**/**0.0663 | 0.0057**/**0.0710 | 0.0037**/**0.0856 |
| m7 | 0.0178**/**0.0845 | 0.0211**/**0.0592 | 0.0245**/**0.0736 | 0.0086**/**0.0511 | 0.0096**/**0.0636 |
| m8‡ | 0.0013**/**0.0162 | 0.0006**/**0.0183 | 0.001**/**0.0119 | 0.0018**/**0.0157 | 0.0014**/**0.0339 |
| m9 | 0.0052**/**0.0179 | 0.0034**/**0.0209 | 0.0031**/**0.0297 | 0.0038**/**0.0296 | 0.0020**/**0.0268 |
| m10 | 0.146**/**0.210 | 0.173**/**0.219 | 0.168**/**0.236 | 0.153**/**0.251 | 0.158**/**0.258 |
| m11 | 0.0052**/**0.0349 | 0.0039**/**0.014 | 0.0074**/**0.0205 | 0.0062**/**0.0311 | 0.0052**/**0.0176 |
| m12 | 0.0555**/**0.349 | 0.0421**/**0.0679 | 0.0384**/**0.0545 | 0.0439**/**0.344 | 0.0186**/**0.0826 |
| m13 | 0.107/0.251 | 0.119/0.220 | 0.115/0.216 | 0.149/0.241 | 0.140/0.239 |
| m14 | - | 0.0020**/**0.0214 | 0.0012**/**0.0230 | 0.0126**/**0.0272 | 0.0178**/**0.0273 |
| m15 | - | - | 0.0402**/**0.0768 | 0.0317**/**0.0602 | 0.0137**/**0.0333 |
| m16 | - | - | - | 0.0056**/**0.114 | 0.0035**/**0.0674 |
| m17 | - | - | - | 0.0337**/**0.0668 | 0.0219**/**0.0555 |
| m18 | - | - | - | 0.0343**/**0.0520 | 0.0223**/**0.0439 |
| m19 | - | - | - | 0.0103**/**0.0826 | 0.0097**/**0.042 |
| m20 | - | - | - | 0.0186**/**0.0398 | 0.0153**/**0.0407 |
| m21 | - | - | - | 0.017**/**0.031 | 0.0118**/**0.0476 |
| m22 | - | - | - | 0.0285**/**0.0469 | 0.0249**/**0.0523 |
| m23 | - | - | - | 0.118**/**0.237 | 0.0533/0.147 |
| m24 | - | - | - | 0.0943**/**0.145 | 0.0658**/**0.112 |
| m25 | - | - | - | 0.0034**/**0.0206 | 0.0072**/**0.0210 |
| m26 | - | - | - | - | 0.0211**/**0.0409 |
| m27 | - | - | - | - | 0.0038**/**0.0422 |
| m28 | - | - | - | - | 0.0046**/**0.0221 |
| m29 | - | - | - | - | 0.0029**/**0.0367 |
| m30 | - | - | - | - | 0.0085**/**0.0661 |
| m31 | - | - | - | - | 0.0026**/**0.0287 |
| m32 | - | - | - | - | 0.0033**/**0.0286 |
| m33 | - | - | - | - | 0.0019**/**0.0167 |

Note:

w, Xu-142 wild-type; m, Xu-142-*fl* mutant;

†, DAPs only detected on gels of the wild-type Xu-142; ‡,DAPs only detected on gels of the mutant Xu-142-*fl*.

-, no significant difference could be detected between wild-type and mutant.
